# Editorial for the Special Issue on AC Electrokinetics in Microfluidic Devices, Volume II

**DOI:** 10.3390/mi15101229

**Published:** 2024-09-30

**Authors:** Antonio Ramos, Pablo García-Sánchez, Raúl Fernández-Mateo

**Affiliations:** Departamento de Electrónica y Electromagnetismo, Facultad de Física, Universidad de Sevilla, Avda, Reina Mercedes s/n, 41012 Sevilla, Spain; pablogarcia@us.es (P.G.-S.); r.fernandez-mateo@soton.ac.uk (R.F.-M.)

The use of AC electric fields in manipulating and characterizing liquids and suspended particles in microfluidic systems continues to drive innovation in several fields, such as colloidal science, microelectronics, and biotechnology. AC electrokinetics has enabled the precise manipulation of biological cells, nanoparticles, and semiconductor materials, allowing for numerous practical applications ranging from diagnostics to materials science. The works presented in this Special Issue show how advancements in AC electrokinetics combined with dielectrophoresis (DEP) within microfluidics have expanded the versatility of particle manipulation and self-assembly and induce liquid operations such as pumping and mixing. Beyond manipulation, AC electrokinetics has been instrumental in characterizing the dielectric properties of particles through DEP. This characterization capability is invaluable in fields such as biosensing and materials science, where understanding particle behavior under electric fields aids in the design of more efficient devices.

The second volume of this Special Issue consist of seven research articles investigating three different areas, which can be grouped into (1) particle/droplet manipulation driven by electric fields combined with fluid flows; (2) fluid flow manipulation, and (3) electrolyte response to electrodes subjected to AC electric fields in microsystems.

1. Miloh and Avital [1] studied theoretical framework models of various electrokinetic phenomena around a conducting and Janus dimer (two touching spheres). They modeled explicit solutions for electro-rotation, traveling-wave DEP or induced-charge electroosmotic (ICEO) flows. Tang et al. [2] studied the rapid oscillatory motion of charged water droplets in oil and on a superhydrophobic surface, achieved through corona discharge. They demonstrated that charge injection provides greater flexibility in controlling droplet movement. Flores-Mena, García-Sánchez, and Ramos [3] investigated how metal colloids scatter around an insulating post in AC fields depending on the particle size, distance, and field frequency. Finally, Gimsa and Radai [4] give an insightful discussion into DEP particle manipulation.
2. Cenaiko, Lijnse, and Dalton [5] explored the performance of electrothermal micropumps, demonstrating that coulombic forces significantly enhance fluid flow rates.
3. López-García, Horno, and Grosse [6] analyzed the AC response of electrolytic cells under DC bias, which reveals a low-frequency dispersion linked to the finite electrode spacing and highlights that a fixed ionic content significantly affects the cells’ steady-state and frequency response, with notable variations in characteristic frequencies under different conditions for closed versus open cells. Tahmasebi et al. [7] investigated how pH gradients affect device performance at various frequencies, revealing that Faradaic reactions increase with lower frequencies and are more prominent in star-shaped microelectrodes. $HfO_{2}$ thin films were also tested, showing frequency-dependent properties that reduce Faradaic reactions.

The diversity of these research papers illustrates the growing importance of AC electrokinetics for microfluidic applications. The ability to manipulate, assemble, and characterize particles at the microscale with electric fields has far-reaching implications for a variety of scientific and industrial sectors. We express our sincere thanks to all contributing authors and reviewers for their efforts in advancing the field. Their work not only enriches our understanding but also inspires future research in AC electrokinetics and microfluidics.
